# Suspected Levetiracetam-Induced Acute Rhabdomyolysis in a Patient With Retinoblastoma: A Case Report and Literature Review

**DOI:** 10.7759/cureus.25183

**Published:** 2022-05-21

**Authors:** Khadija M Alshehabi, Sumayah Askandarani, Zainab A Alkhalifah

**Affiliations:** 1 Nephrology, Salmaniya Medical Complex, Manama, BHR; 2 Nephrology, King Fahad Specialist Hospital, Eastern Health Cluster, Dammam, SAU; 3 Internal Medicine, King Fahad Specialist Hospital, Eastern Health Cluster, Dammam, SAU

**Keywords:** levetiracetam, hemodialysis, retinoblastoma, acute kidney injury, rhabdomyolysis

## Abstract

Rhabdomyolysis is a condition characterized by the destruction of the skeletal muscle and the release of its content into the circulation, and it can cause acute kidney injury (AKI). There are numerous causes for the development of this condition, and some of them are rare. Levetiracetam, an antiepileptic agent, has been speculated as a rare possibility for the development of rhabdomyolysis. In this report, we highlight a case of a 36-year-old gentleman with retinoblastoma since childhood, who was maintained on levetiracetam for two years for epilepsy. He was brought to our hospital with a history of generalized fatigue and unwitnessed seizure. Upon further investigations, he was found to have severe rhabdomyolysis and AKI that required renal replacement therapy. Levetiracetam was suspected as a culprit and therefore was discontinued with gradual improvement of renal function over a few months.

## Introduction

Levetiracetam is a second-generation antiepileptic drug that has been approved worldwide for treating partial and generalized seizures. Levetiracetam acquires unique mechanisms of action that involve binding to synaptic vesicle protein 2A, inhibiting calcium release from intraneural stores, and eventually inhibiting the excessive activity between the neurons [1]. There are various adverse effects of this medication, some of which include somnolence, headache, asthenia, accidental injury, dizziness, infection, flu-like syndrome, vomiting, diarrhea, and behavioral effects [2]. Yet, few publications correlated levetiracetam and the occurrence of rhabdomyolysis.

In our case, we will discuss the development of acute rhabdomyolysis as a rare side effect of levetiracetam. We have found 14 case reports in the literature examining the association between levetiracetam and rhabdomyolysis [3].

## Case presentation

This 36-year-old gentleman was diagnosed with retinoblastoma in childhood, for which he received radiotherapy to his right eye while his left eye was resected. He became blind at the age of five. He also developed episodes of generalized tonic-clonic convulsions in 2018. An MRI of the brain showed right orbital-sphenoidal wing atypical meningioma, which was resected and followed by local radiotherapy. Since then, he was kept on levetiracetam 500 mg twice per day and has followed up with our hospital's neurology team.

On June 21, 2020, the patient was brought by his father to the emergency department with a four-day history of generalized body fatigue. It was proceeded by an episode of unwitnessed seizure as his mother found him lying on the ground, and his clothes were covered with vomitus and stool, yet he was fully conscious.

At the emergency department, he was fully conscious and oriented to time, person, and place but looked dehydrated. His vital signs were maintained, except that his blood pressure was on the lower side at 90/50 mmHg. The patient was noted to be anuric. His physical examination was unremarkable. When reviewing his investigations, his renal function was significantly impaired with evidence of hyperkalemia, hyperphosphatemia, hypocalcemia, severe acidosis, and raised creatinine to 1185 mmol/L from a baseline of 95 mmol/L. His toxicology screen was negative and a brain CT was unremarkable. It was also noticed that the patient's creatine kinase-MB (CK-MB) was markedly elevated, reaching > 853,400 U/L. The patient was admitted under the care of nephrology with the impression of acute kidney injury (AKI) secondary to rhabdomyolysis.

**Figure 1:**
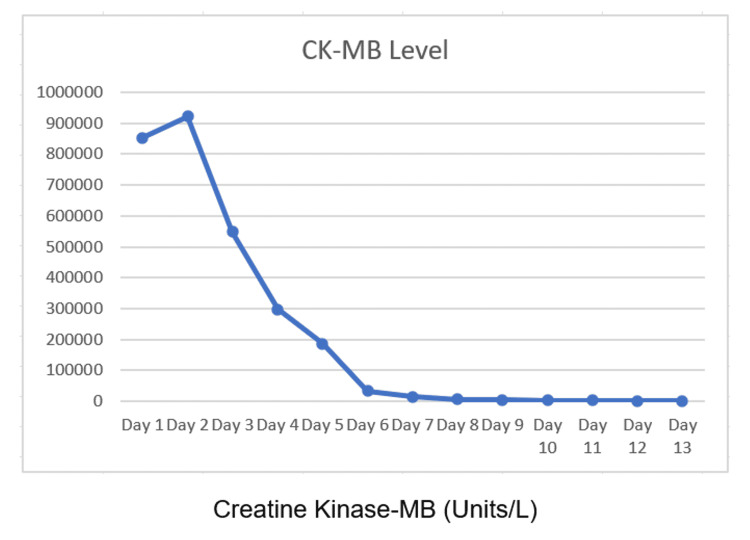
Trend of creatine kinase-MB (CK-MB) during hospitalization

Attempts of aggressive hydration failed to improve the patient's renal function or urine output over 24 hours, so the decision of initiating renal replacement therapy (RRT) in the form of hemodialysis was made. The patient was started on conventional intermittent hemodialysis with close monitoring of his vital signs and laboratory parameters, including renal function test, CK-MB, and bone profile. Furthermore, all efforts were made to look for the causes of rhabdomyolysis. While an episode of convulsion with prolonged immobilization could be on top of the list, the patient's CK-MB continued to rise, reaching > 921,672 U/L despite aggressive hydration and daily hemodialysis. This event triggered us to search for hidden or uncommon causes for the patient's severe rhabdomyolysis, and here, we made a correlation between his antiepileptic medication "levetiracetam" as a possible cause for his rhabdomyolysis and AKI since few case reports were published in this regard. Therefore, levetiracetam was replaced with carbamazepine after consulting the neurology team, and a few days later, the patient's investigations started to improve gradually, including his CK-MB, which began to decrease (Figure 1).

The patient was discharged and maintained on regular hemodialysis for two months until August 2020, when his kidneys showed signs of renal recovery and the latest creatinine reached 176 mmol/L on December 13, 2020.

## Discussion

Rhabdomyolysis is a clinical syndrome associated with injury to the skeletal muscle fibers and the release of intracellular contents into the systemic circulation [4,5]. These contents are considered the markers of muscle damage and necrosis and consist of myoglobin, creatine phosphokinase, and lactate dehydrogenase [4,6]. This condition was originally described by Bywater and Bell in four cases of crush injuries during the bombing of London in World War II [6].

Rhabdomyolysis can be severe enough to cause AKI due to heme pigment nephropathy. The etiologies are diverse, including trauma, crush injuries, medications such as statins, toxins, excessive exercise, electrolyte disturbance, inflammatory conditions such as vasculitis, and hereditary and acquired metabolic myopathies [5,6]. However, several case reports described levetiracetam as a rare adverse event for developing this syndrome.

Levetiracetam is a second-generation antiepileptic medication approved worldwide to treat generalized and partial seizures with an acceptable adverse effects profile and few significant side effects beyond somnolence, infection, and flu syndrome [1,2]. Nonetheless, when exploring the literature, we have found 14 case reports about levetiracetam-induced rhabdomyolysis since 2014, with the latest one published in 2020 [3]. It was observed that all the reported cases, including ours, were of young patients with ages ranging from 16 to 42 years old and with male predominance [3]. Only one case was of a patient above 60 years [7]. Of the 14 cases, only five reported AKI but without requiring RRT [5,8-11]. Our case would be the first to report severe rhabdomyolysis, possibly secondary to levetiracetam and the development of AKI requiring hemodialysis with a slow renal recovery pattern over two months. Our patient was the only one on levetiracetam for a considerable period of time, i.e., around two years. He was tolerating it well and never complained of any specific side effects.

## Conclusions

Despite the rare correlation, levetiracetam can potentially cause rhabdomyolysis leading to AKI. The underlying mechanism by which levetiracetam causes acute rhabdomyolysis is still unknown, and further studies are necessary to empower this relationship. Yet, clinicians should be attentive to this rare side effect of levetiracetam not merely on newly initiated patients but even on those taking it for an extended period, like our patient.
